# The impact of environmental factors on human echinococcosis epidemics: spatial modelling and risk prediction

**DOI:** 10.1186/s13071-022-05169-y

**Published:** 2022-02-08

**Authors:** Jie Yin, Xiaoxu Wu, Chenlu Li, Jiatong Han, Hongxu Xiang

**Affiliations:** Present Address: State Key Laboratory of Remote Sensing Science, College of Global Change and Earth System Science, Beijing Normal University, 19 Xinjiekouwai Street, Haidian District, Beijing, 100875 China

**Keywords:** Echinococcosis, Natural environment, Hot spot, Model, Spatial prediction

## Abstract

**Background:**

Human echinococcosis is affected by natural environmental factors, and its prevalence shows a distinct geographical distribution. Western China has the highest endemicity of human echinococcosis worldwide, but the spatial pattern and environmental determinants of echinococcosis are still unclear.

**Methods:**

Hot/cold spot analysis was used to investigate the spatial distribution of human echinococcosis prevalence. Geodetector was used to identify key natural factors, and a structured additive regression model was used to analyse the relationship between natural factors and human echinococcosis prevalence and spatially predict echinococcosis epidemics.

**Results:**

Hot spots for human echinococcosis prevalence include western and southeastern parts of Tibet Autonomous Region (henceforth Tibet) and the border areas between Tibet and the provinces of Qinghai and Sichuan. Spatial effects are crucial when modelling epidemics, and relative humidity, altitude and grassland area ratio were found to have the most evident effects on echinococcosis epidemics. The relationship between these three factors and echinococcosis prevalence was non-linear, and echinococcosis risk was higher in areas with high relative humidity, high altitude, and a high ratio of grassland to other land use types. The prevalence that was predicted from the investigated environmental factors was generally higher than the actual prevalence, and more epidemic hot spots were predicted for the Qinghai-Tibet Plateau, Inner Mongolia Autonomous Region, and the provinces of Yunnan and Sichuan than the rest of western China. These results indicate that prevention and control measures may effectively reduce echinococcosis prevalence.

**Conclusions:**

We suggest that the prevention and control of human echinococcosis should be prioritized in the hot spots identified here, through the rational allocation of limited medical resources to where they are most needed. Furthermore, the spatial epidemiological modelling methods used in this study can be employed in future studies on echinococcosis and similar diseases.

**Graphical abstract:**

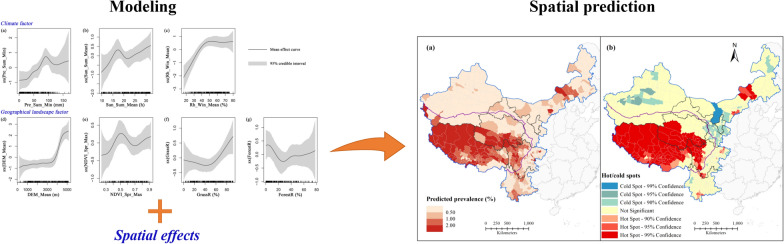

**Supplementary Information:**

The online version contains supplementary material available at 10.1186/s13071-022-05169-y.

## Background

Human echinococcosis is a parasitic disease caused by the larval stages of species of the genus *Echinococcus*. The two most important forms of echinococcosis in humans are cystic echinococcosis (CE) and alveolar echinococcosis (AE), which are caused by the tapeworms *Echinococcus granulosus* and *Echinococcus multilocularis*, respectively. The annual global disease burdens of CE and AE are 1 million and 666,000 disability-adjusted life years, respectively [1, 2]. Both CE and AE have a distinctive geographical distribution [3]. CE is globally distributed except for Antarctica; parts of Eurasia, northern and eastern Africa, Australia and South America are hyperendemic for this disease [4]. In contrast, AE is only found in the northern hemisphere, and especially in China, Russia, Europe and North America [3]. China has a disease burden of 40% and 95% of the global CE and AE disability-adjusted life years, respectively [1, 5]. Human echinococcosis is a huge burden on health in China, especially in economically disadvantaged agricultural and pastoral areas. The distinct geographical distribution of human echinococcosis implies that it is closely related to natural environmental factors, thus it was considered important to investigate how the latter impact echinococcosis epidemics.

Natural environmental factors can affect the transmission of echinococcosis to humans. The survival of *Echinococcus* spp. eggs, the population dynamics and spatial distribution of the hosts of *Echinococcus*, and human exposure risk are all directly or indirectly affected by natural environmental factors [6]. Natural risk factors for echinococcosis fall under two categories: climate and geographical landscape. Temperature (T), precipitation (Pre), and relative humidity (Rh) are the main climate risk factors for echinococcosis. In France, the risk of AE is higher in areas with very cold winters and high annual Pre [7]. CE prevalence was found to be negatively correlated with land surface temperature in China [8, 9], and nonlinearly related to annual mean Pre in the Tibet Autonomous Region (henceforth Tibet) [10]. Altitude, land use type, vegetation type, and landscape pattern are the main geographical landscape factors related to echinococcosis prevalence. Two studies found that altitude is positively correlated with echinococcosis prevalence [8, 10]. Another study indicated that, in China, AE risk is higher in high-altitude alpine grassland areas [11]. As echinococcosis prevalence is positively correlated with grassland area ratio and negatively correlated with forest area ratio [9, 11], the primary focus of the present study was these two natural environmental factors.

Although the natural environmental factors that affect human echinococcosis prevalence have been analysed in many studies, most of these were focussed on the relationship between specific natural factors and human echinococcosis, and there has been no research on natural environmental factors as predictors of echinococcosis epidemic risk. In the present study, hot/cold spots and natural risk factors for human echinococcosis (both CE and AE) prevalence were analysed, and an optimal county-level model including spatial effects developed for western China. In addition, using this model, the relationships between key natural factors and human echinococcosis were analysed and the prevalences and potential hot/cold spots of human echinococcosis across western China predicted.

## Methods

### Study area

**Fig. 1:**
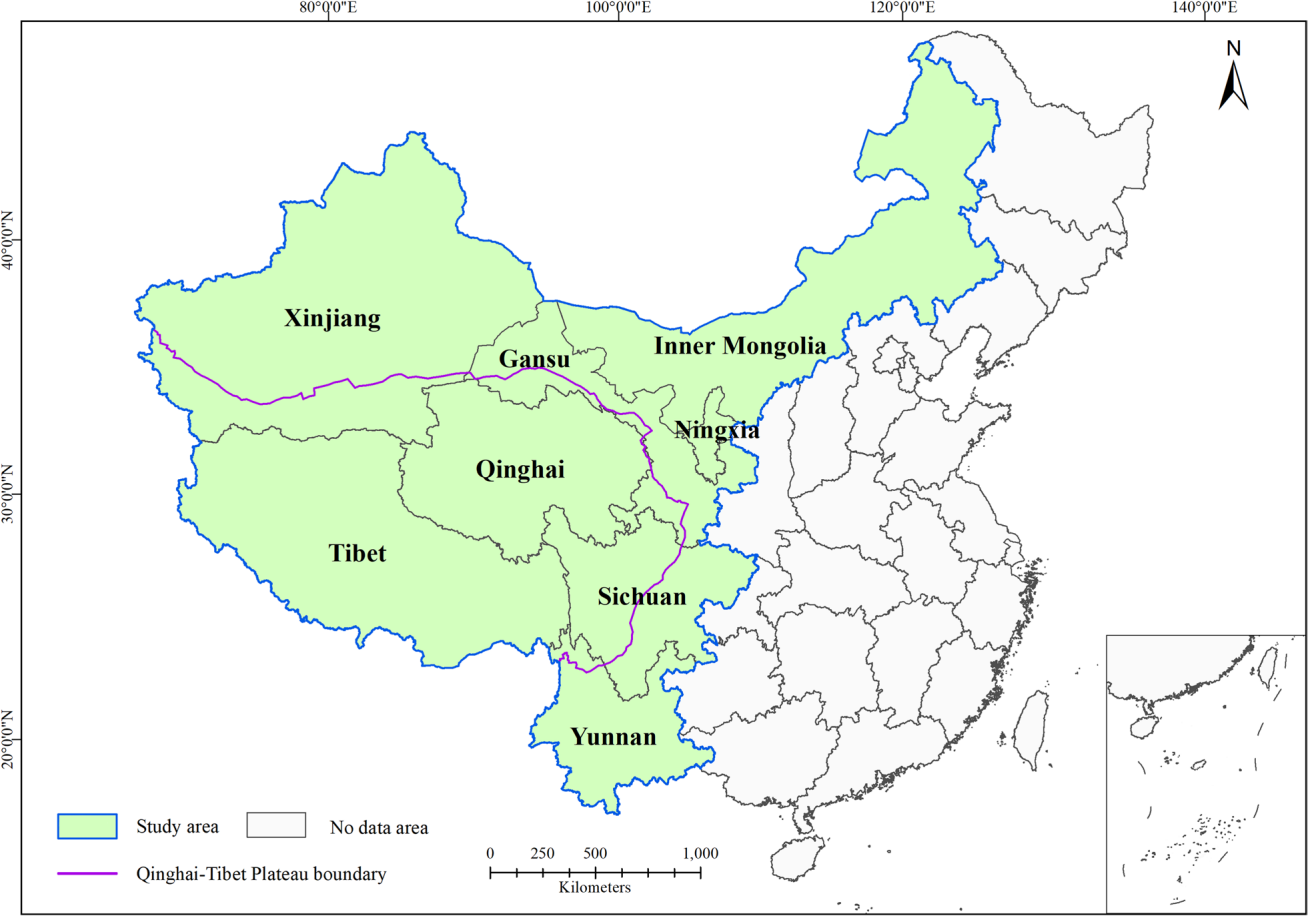
Map of the study area

Western China has a high endemicity of echinococcosis in the world [12]. Therefore, 344 counties of western China were selected as the study area for this research. These counties are located in the Inner Mongolia Autonomous Region (henceforth Inner Mongolia), the Ningxia Hui Autonomous Region (henceforth Ningxia), the provinces of Gansu, Qinghai, Sichuan and Yunnan, the Xinjiang Uygur Autonomous Region (henceforth Xinjiang), and Tibet (Fig. 1). These areas are the most endemic for echinococcosis in China, and their natural environmental conditions are diverse. The southwestern study area has high rainfall and abundant animal and plant resources; the northwestern study area is arid and rainless, with relatively high levels of sunshine; and the Qinghai-Tibet Plateau is high in altitude and experiences low temperatures.

### Data collection and preprocessing

The county-level prevalence data of human echinococcosis were collected from recent scientific papers and reports. The data for 24 counties in Yunnan and 70 counties in Tibet were derived from papers published by local centers for disease control [13–20], and the data for the remaining 150 counties were derived from epidemiological surveys [21]. The same methodology was used for all the epidemiological surveys carried out in these counties, and the details of the diagnosed cases and human prevalence estimates are described in Wang [21].

In the present study, nine natural environmental factors, which fall into two categories, climate and geographical landscape, and a total of 81 variables, were considered (Table 1). The climatic factors comprised T, Pre, Rh, and sunshine duration (Sun). The geographical landscape factors comprised a digital elevation model (DEM), the normalized difference vegetation index (NDVI), proportion of grassland to total land use area (GrassR), proportion of forest to total land use area (ForestR), and proportion of cultivated land to total land use area (CultivatedR). Statistical indices included minimum (Min), maximum (Max), and mean values (Mean). The 81 variables comprised GrassR, ForestR, CultivatedR, minimum DEM (DEM_Min), maximum DEM (DEM_Max), and mean DEM (DEM_Mean), and 75 other variables, which included T, Pre, Rh, Sun, and NDVI extracted from seasonal and statistical indices. Seasonal indices included spring (Spr), summer (Sum), autumn (Aut), and winter (Win). The naming rule for the variables was factor_seasonal index_statistical index. For example, Pre_Sum_Min represents minimum precipitation in summer. The temporal information for the epidemiological surveys and all of the 81 variables are given in Additional file 1: Tables S1 and S2.

**Table 1:** Attributes of the natural environmental factors

Category	Factor	Description	Data source
Climate	T	Temperature	National Earth System Science Data Center, National Science & Technology Infrastructure of China (http://www.geodata.cn)
	Pre	Precipitation	
	Rh	Relative humidity	
	Sun	Sunshine duration	
Geographical landscape	DEM	Digital elevation model	Resource and Environment Data Cloud Platform (http://www.resdc.cn/DataList.aspx)
	NDVI	Normalized difference vegetation index	
	GrassR	Proportion of grassland to total land use area	
	ForestR	Proportion of forest to total land use area	
	CultivatedR	Proportion of cultivated land to total land use area	

### Analytical methods

The Getis-Ord Gi* statistic was used to identify statistically significant hot/cold spots. The spatial clustering of high/low values (hot/cold spots) was obtained by calculating and analysing the* z*-scores and* p*-values. The higher (or lower, negative) the* z*-score, the more intense the clustering. A* z*-score near zero indicated no apparent spatial clustering. A high (or low, negative)* z*-score and small* p*-value for a feature indicated spatial clustering of high (or low) values. All the analyses were conducted using the hot spot analysis tool in ArcGIS 10.3.1.

Geodetector is used to detect spatially stratified heterogeneity and determine driving factors [22, 23]. In this study, Geodetector was used to detect the contribution of each factor (*X*) to human echinococcosis prevalence (*Y*) according to the * q*-statistic value. The* q*-statistic is defined by the following equation:

1 $${\text{q}} = 1 - \frac{{\mathop \sum \nolimits_{h = 1}^{L} N_{h} \sigma_{h}^{2} }}{{N\sigma^{2} }}$$

where *N* is the number of samples in the study area, *L* is the number of categories of* X*, $\sigma^{2}$ is the total variance of* Y* in the study area, and $\sigma_{h}^{2}$ is the variance of* Y* within category *h* of* X*. The larger the* q*-value, the better that* X* explains* Y*.

A structured additive regression (STAR) model was used to assess the natural determinants of echinococcosis prevalence. In this study, the STAR model was estimated using Bayesian inference. The penalized least squares approach was used to select relevant covariates after Geodetector analysis, and a fully Bayesian estimation based on the Markov chain Monte Carlo method was used for model estimation. As echinococcosis prevalence is spatially heterogeneous, two models were developed that differ in whether or not they include spatial effects for comparison. The spatial effect term described the geographical spatial relationship for each county. These models are given by:

2 $${\text{Model}}\,{\text{I}}:prevalence_{i} = \alpha_{0} + \mathop \sum \limits_{k = 1}^{q} f_{k} \left( {x_{ik} } \right)$$

3 $${\text{Model}}\,{\text{II}}:prevalence_{i} = \alpha_{0} + \mathop \sum \limits_{k = 1}^{q} f_{k} \left( {x_{ik} } \right) + f_{unstr} \left( {County_{i} } \right)$$

where $prevalence_{i}$ is the prevalence of echinococcosis in county $i \left( {i = 1, \ldots n} \right)$; $\alpha_{0}$ is the intercept; $x_{ik} = \left( {x_{i1} , \ldots x_{iq} } \right)$ is a vector containing *q* covariates; $f_{k} \left( {x_{ik} } \right)$ is the nonlinear smooth functions of the covariates $x_{ik}$, where Bayesian P-splines are utilized; $f_{unstr} \left( {County_{i} } \right)$ is a function that accounts for unstructured spatial effects for each county, where we define a Markov random field prior for a spatial covariate.

The deviance information criterion (DIC) value was used for model checking and comparison; usually the model with the smallest DIC is the preferred one. The DIC was calculated as $DIC = { }\overline{D} + 2p_{D}$, where $\overline{D}$ is the posterior mean of the deviance, which is a measure of how well the model fits the data, and $p_{D}$ is the effective number of parameters, which is a complexity penalty on the model. The root-mean-square error (RMSE) and residual predictive deviation (RPD) were used to compare reported prevalence and model-fitted prevalence, which can measure the predictive ability of the model. Fully Bayesian analyses were conducted using the R package R2BayesX.

At the factor selection and modelling analysis stage, the time that all natural data were recorded was consistent with the time that human echinococcosis prevalence was surveyed for each county. In the modelling and predictive stage, the natural data from 2016 were used for all the counties in western China. The temporal information for the data is given in Additional file 1: Table S2.

## Results

### Spatial distribution of human echinococcosis

**Fig. 2:**
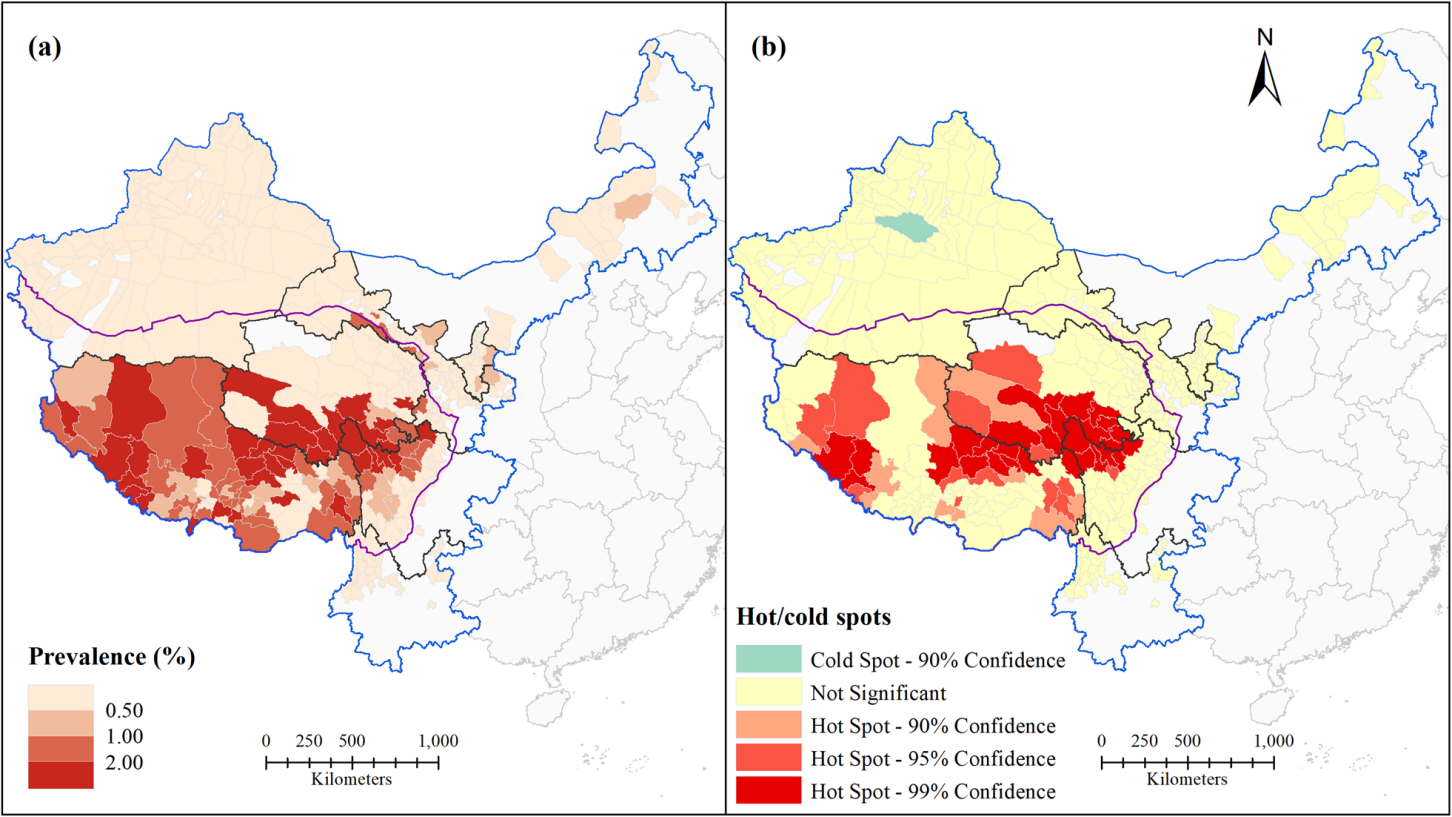
Spatial distribution of echinococcosis at the county level in western China: **a** echinococcosis prevalence; **b** hot/cold spots

Figure 2a shows the spatial distribution of human echinococcosis prevalence in western China. Human echinococcosis is endemic in eight provinces/regions of western China, and Qinghai, Sichuan, and Tibet are those with the highest endemicity. The distribution of human echinococcosis prevalence in western China shows spatial clustering. Highly endemic areas are mainly concentrated on the Qinghai-Tibet Plateau, especially the southwestern part, where human echinococcosis prevalence is highest. Although human echinococcosis is also endemic in counties that are not part of the Qinghai-Tibet Plateau, the prevalences in most of these is generally low (less than 0.5%). Western and southeastern parts of Tibet and the border areas of Tibet, Qinghai, and Sichuan were hot spots (Fig. 2b). A cold spot was determined in northwestern Xinjiang, and the prevalences in other counties of this region did not show statistically significant spatial clustering. All three hot spots were located on the Qinghai-Tibet Plateau, which indicates that environmental conditions there are suitable for human echinococcosis transmission. The results indicate that unstructured spatial effects may be potential risk factors for human echinococcosis.

## Modelling of natural factors and human echinococcosis

### Identification of key natural factors

**Fig. 3:**
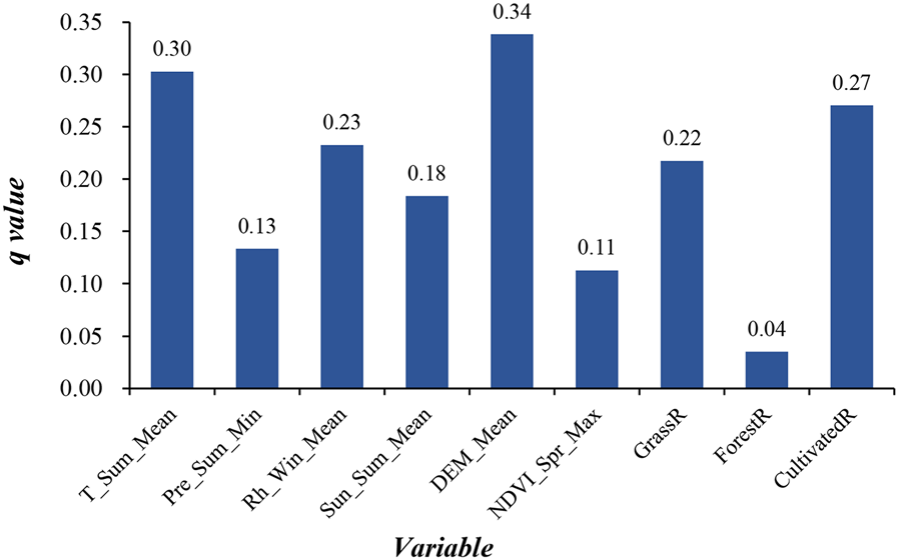
The* q*-values of variables based on the Geodetector analysis.* T* Temperature,* Sum* summer,* Mean* mean value,* Pre* precipitation,* Rh* relative humidity,* Win* winter,* Sun* sunshine duration,* DEM* digital elevation model,* NDVI* normalized difference vegetation index,* Spr* spring,* Max* maximum,* GrassR* proportion of grassland to total land use area,* ForestR* proportion of forest to total land use area,* CultivatedR* proportion of cultivated land to total land use area

Key natural risk factors were identified from the 81 variables examined using Geodetector. All the selected factors had the largest* q*-value of each type, which means that they contributed greatly to human echinococcosis prevalence and could be considered for inclusion in the modelling. Figure 3 shows the nine selected factors and their contribution to human echinococcosis prevalence. These latter factors were used as input for the STAR model for the final selection of factors using the penalized least squares approach. Finally, Pre_Sum_Min, Rh_Win_Mean, Sun_Sum_Mean, DEM_Mean, NDVI_Spr_Max, GrassR, and ForestR were included in the modelling analysis.

### Model comparison and validation

Using human echinococcosis prevalence as the dependent variable and the seven selected key factors as the independent variables, two STAR models were developed for comparison. The first model (model I) included the selected key factors, and the second model (model II) considered the spatial random effect based on model I. DIC, RMSE, and RPD were used to compare the models, and the results are given in Table 2. The predictive ability and fit of model II were notably better than those of model I, which indicated that the spatial random effect was essential for modelling human echinococcosis prevalence with respect to natural environmental factors.

**Table 2:** Comparison of model fit

Model fit	Model I	Model II
$\overline{D}$	964.50	507.37
*p*_*D*_	38.84	− 13.60
DIC	1042.17	480.17
RMSE	0.98	0.43
RPD	1.53	3.53

*p*_*D*_ Effective number of parameters, *DIC* deviance information criterion,* RMSE* root-mean-square error,* RPD* residual predictive deviation

**Fig. 4:**
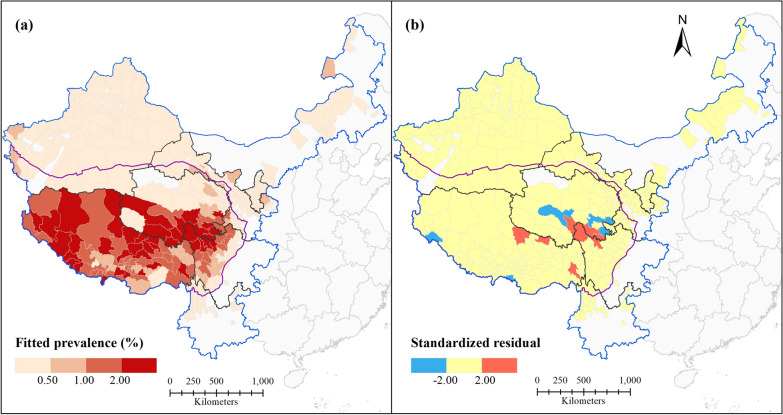
Fit and evaluation of model II: **a** fitted prevalence; **b** standardized residual

The fit of model II was validated by the RMSE value and by comparing the reported values (Fig. 2a) and the fitted values (Fig. 4a) of human echinococcosis prevalence. The results demonstrated that model II fits well the prevalence and spatial distribution pattern of human echinococcosis in western China. The model was considered qualified as 95.95% of the standardized residuals (Fig. 4b) were in the interval (− 2, 2).

### Analysis of the relationships between key natural factors and human echinococcosis

**Fig. 5:**
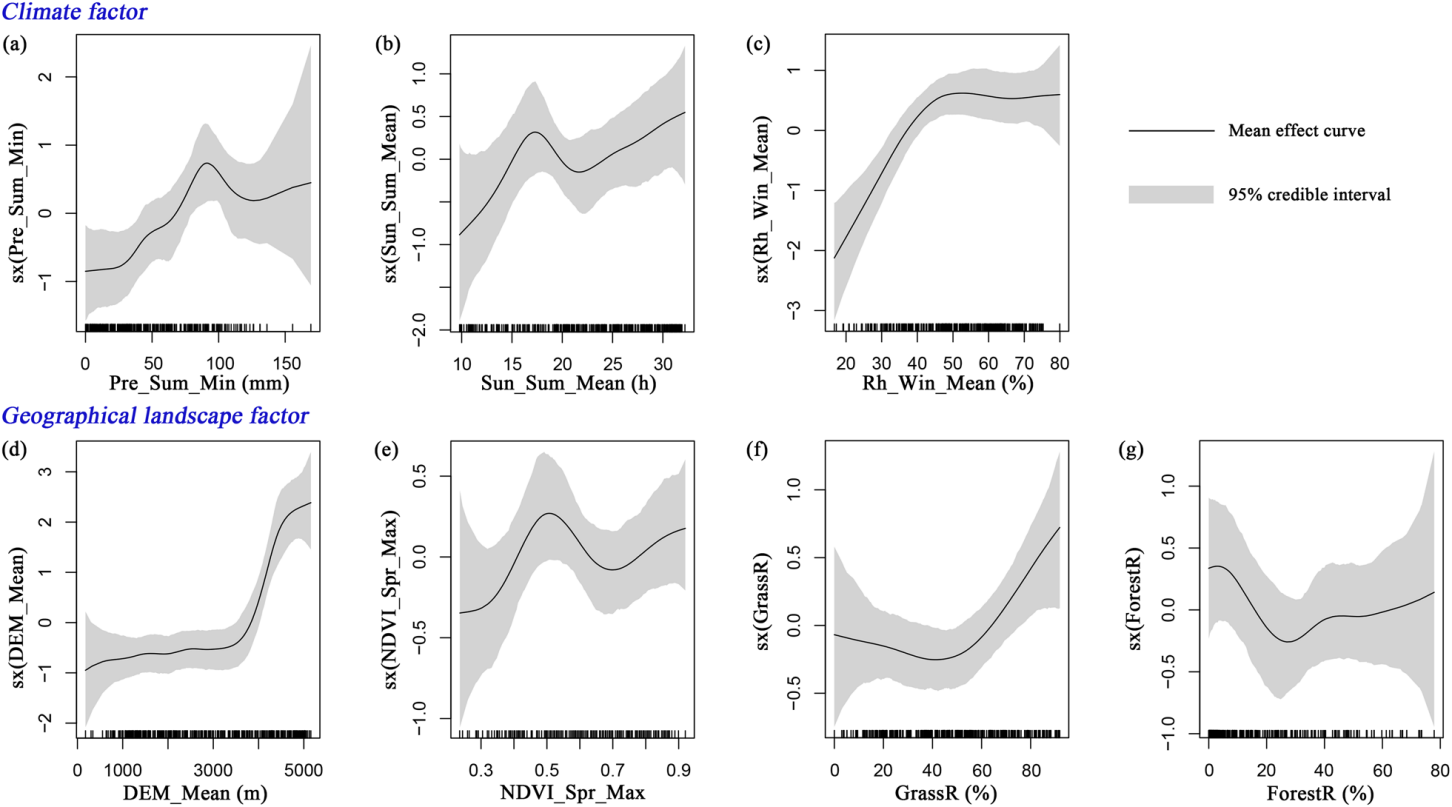
Nonlinear relationships between key natural factors and human echinococcosis prevalence: **a** Pre_Sum_Min; **b** Sun_Sum_Mean; **c** Rh_Win_Mean; **d** DEM_Mean; **e** NDVI_Spr_Max; **f** GrassR; **g** ForestR. For abbreviations, see Fig. 3

The nonlinear effects of the investigated key natural factors on human echinococcosis prevalence are shown in Fig. 5, together with the 95% credible intervals. Human echinococcosis prevalence was affected by climate factors, including Pre, Sun, and Rh. Human echinococcosis prevalence increased with Pre_Sum_Min and Sun_Sum_Mean when they were below 90 mm and 17.5 h, respectively (Fig. 5a, b). With a further increase in these factors, prevalence first decreased and then increased. Human echinococcosis prevalence gradually increased with Rh_Win_Mean, reached a peak when it was 50%, and then stabilized (Fig. 5c). Geographical landscape factors also had a clear impact on human echinococcosis prevalence, especially altitude and vegetation characteristics. Figure 5d shows the steady rise in human echinococcosis prevalence with increasing DEM_Mean. A lower prevalence was observed when DEM_Mean was below 3500 m, while the prevalence increased sharply when DEM_Mean was higher than 3500 m. Human echinococcosis prevalence gradually increased when NDVI_Spr_Max was below 0.5 or above 0.7, and decreased when it was within the range 0.5–0.7 (Fig. 5e). Human echinococcosis prevalence was lower when GrassR was below 50%, and increased rapidly thereafter (Fig. 5f). Although human echinococcosis prevalence varied with ForestR (Fig. 5g), there was no significant correlation between them.

### Spatial prediction of human echinococcosis prevalence

**Fig. 6:**
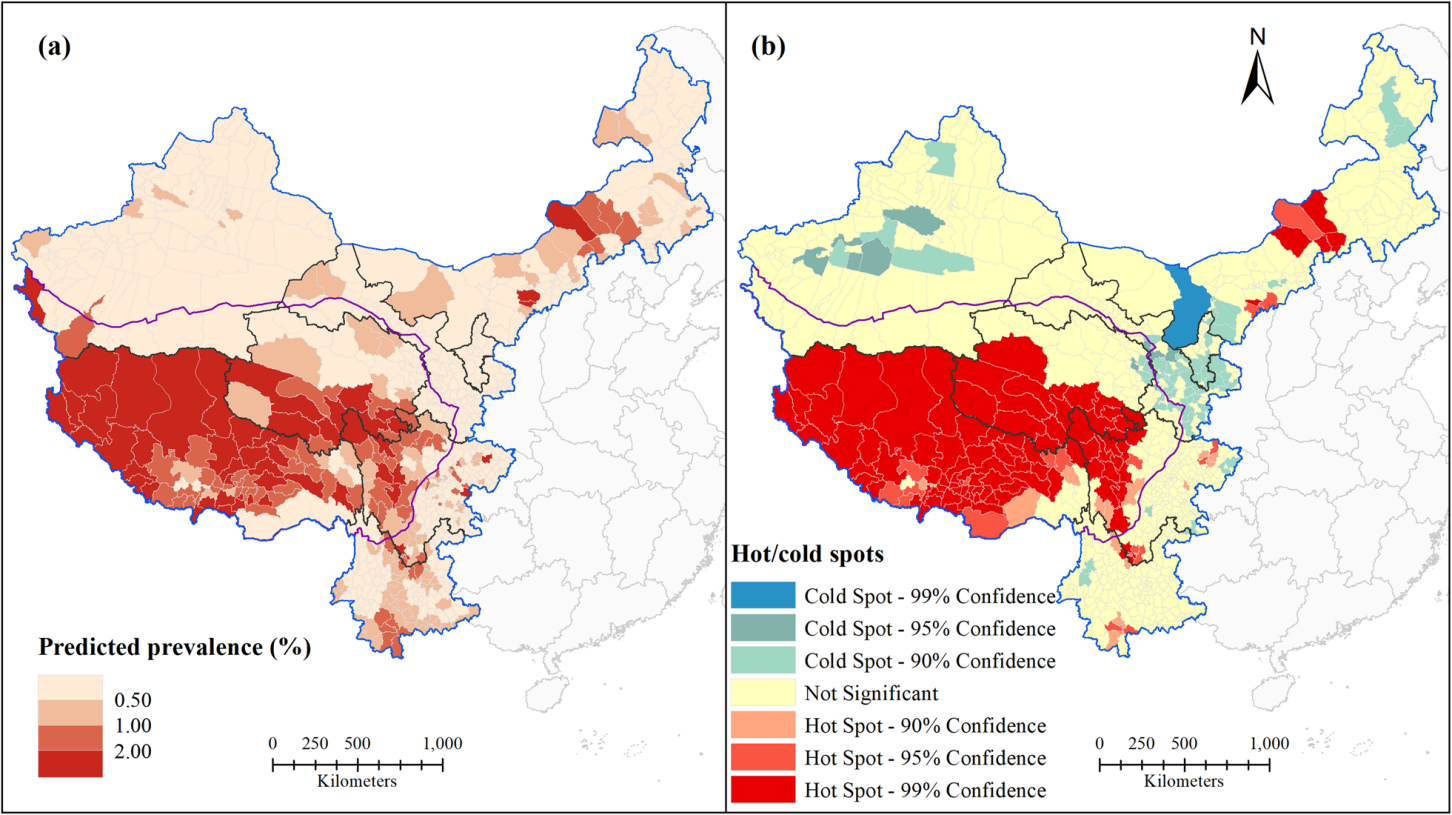
Predicted spatial distribution of human echinococcosis at the county level in western China: **a** predicted prevalence; **b** predicted hot/cold spots

Key natural factor data for 2016 were used to predict the prevalence and hot/cold spots of human echinococcosis in 743 counties of western China in model II (Fig. 6). Figure 6a shows the geographic distribution of predicted human echinococcosis prevalence in each county of western China. The predicted human echinococcosis prevalences based on natural environmental factors were higher than the actual prevalences. For the Qinghai-Tibet Plateau, which has a high prevalence, the predicted prevalence was generally higher than the surveyed one, especially in the case of Tibet. Clusters of higher prevalence were predicted for some areas with relatively low endemicity for human echinococcosis, e.g. central Inner Mongolia, eastern and southern Sichuan, and southern Yunnan. The hot spot analysis also revealed natural risk for human echinococcosis epidemics in western China (Fig. 6b). Compared with the results of the hot spot analysis of the reported prevalences (Fig. 2b), there was more spatial clustering in the predicted prevalences, and more hot and cold spots were observed. More hot spots were observed especially in Tibet and the Tibetan areas of Qinghai and Sichuan. Some scattered hot spots were also observed in Inner Mongolia, Sichuan, and Yunnan. Cold spots were observed in all areas except for Tibet, and there were more cold spot clusters in Inner Mongolia, Ningxia, Gansu, and Xinjiang. These phenomena indicated that the natural environmental factors examined play a role in the transmission and prevalence of human echinococcosis in western China. Thus, more effective targeted prevention and control measures need to be implemented in these areas.

## Discussion

The impact of two types of natural environmental factors on the prevalence of human echinococcosis was investigated. Climate factors have a significant effect on human echinococcosis [6, 11]. Our study indicated that Pre, Sun, and Rh are three important climate factors for human echinococcosis. The release, survival, and infectivity of *Echinococcus* eggs are sensitive to climatic factors, especially humidity [24–26]. One study [25] showed that a dry environment was inimical for echinococcosis transmission. A study in western China [9] found that CE prevalence was significantly positively correlated with annual mean Pre. A significant positive correlation between human seropositivity for *E. granulosus* and *E. multilocularis* and summer Pre was reported for Ningxia [27].

Geographical landscape factors have been reported to be important drivers for the transmission of echinococcosis [11]. Two studies [6, 28] showed that the spatial overlap and predation of the definitive host on the intermediate host were related to landscape factors, which directly affected the transmission of echinococcosis. Two studies [10, 11] found that echinococcosis prevalence was positively correlated with altitude, especially in highly endemic areas in China, which were concentrated at high altitudes. One of these studies [11] showed that AE prevalence was positively correlated with alpine meadows and negatively correlated with forests in mainland China. CE prevalence was significantly positively correlated with NDVI in winter in western China [9].

**Fig. 7:**
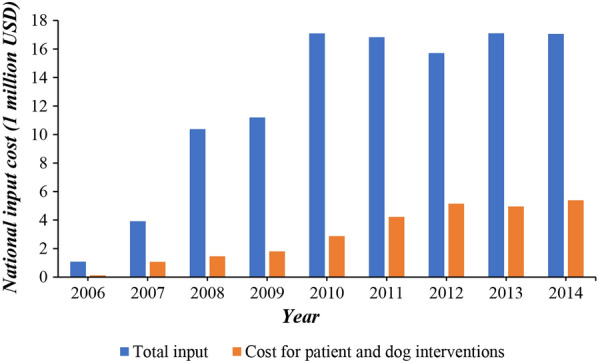
Total expenditure and expenditure on interventions aimed at humans and dogs of the Chinese national control program for echinococcosis, 2006–2014

In the present study, the predicted prevalence was overestimated. Although there may be many reasons for this, the most important one is thought to be the efficacy of the national control program for echinococcosis which was initiated by the Chinese government in 2006 [29]. After the completion of the national program, human echinococcosis prevalence had decreased from 1.08%, in 2004, to 0.28% in 2016 [12, 30]. Compared with 2006, by 2014 the annual total expenditure for the control of echinococcosis and specifically that for interventions aimed at humans and dogs had increased 15.76- and 45.65-fold, respectively (Fig. 7) [31]. Health education, improvements in sanitation, and interventions targeted at humans (ultrasound screening, surgical and albendazole treatment) and dogs (management and deworming) were the main measures implemented [29, 32]. The outcomes show that strengthening preventative and control measures is decisive for the mitigation of echinococcosis epidemics. Although the Chinese government prioritizes the prevention and control of human echinococcosis, the current levels of expenditure on these are not sufficient for the treatment of new patients and the deworming of dogs [31]. Our spatial prediction results are meaningful for the rational allocation of limited resources as they indicate specific potential hot spots where medical resources should be prioritized.

To the best of our knowledge, this study is the first to model and predict the spatial distribution of echinococcosis in western China, where the prevalence of this disease is the highest in the world. The methodology used in the present study improves on that used in previous studies in three ways. First, this is the first study to systematically screen and model natural factors impacting human echinococcosis in western China, and a more comprehensive range of important factors was considered than in previous studies. Second, spatial differences between each county were taken into consideration in the modelling analysis, which significantly improved the accuracy of the model fit. Third, we predicted human echinococcosis prevalence spatially based on natural environmental factors and spatial effects, and hot/cold spots of human echinococcosis prevalence were mapped.

However, there were a few inevitable limitations to this study, which should be acknowledged. First, the epidemiological surveys were not conducted at the same time in each of the studied counties. Second, some additional risk factors for human echinococcosis were not considered in this study. For example, socioeconomic conditions, demographic characteristics, religious beliefs, human behaviour and habits, and host animal infections, which can also affect the prevalence of human echinococcosis. Some studies [9–12] found that gross domestic product, Tibetan population rate, and number of yaks and dogs were all key risk factors for the transmission and prevalence of echinococcosis in western China. Due to data limitations, it was difficult to achieve comprehensive spatial predictions based on natural, social and other factors in the present study. Although the predicted prevalences based on the natural environmental factors were overestimates, they do represent a relative risk for human echinococcosis epidemics. At the same time, they also reflect the importance of implementing measures for the control of echinococcosis. Therefore, in our future research we will also estimate more comprehensively, at a smaller spatial scale, the effects of natural environmental factors, socioeconomic conditions and human interventions on echinococcosis prevalence.

## Conclusions

We investigated the effects of natural environmental factors on human echinococcosis at the county level in western China. Hot spots of human echinococcosis prevalence were mainly concentrated on the Qinghai-Tibet Plateau, especially in the western and southeastern parts of Tibet and the border areas of Tibet, Qinghai, and Sichuan. Pre_Sum_Min, Rh_Win_Mean, Sun_Sum_Mean, DEM_Mean, NDVI_Spr_Max, GrassR, and ForestR were the key natural factors identified from the modelling. The results of the modelling showed that the spatial effect was crucial, and that humidity, altitude, and grassland area ratio have a clearer relationship with human echinococcosis epidemics than the other factors investigated. The predicted prevalences revealed that some natural environmental factors are favourable for the transmission of human echinococcosis in western China and epidemics of this disease. Almost the entire Qinghai-Tibet Plateau was a hot spot for human echinococcosis prevalence, indicating that more attention needs to be paid to echinococcosis there and resources targeted for its prevention and control. Although the Chinese government prioritizes the prevention and control of human echinococcosis, the widespread epidemic of this disease in China indicates that more medical resources are required for its treatment. Therefore, we suggest that counties of the Qinghai-Tibet Plateau, Inner Mongolia, Yunnan and Sichuan, all of which had hot spots, should be prioritized for targeted prevention, control and long-term monitoring of human echinococcosis. This study provides an excellent model for investigating relationships between environmental factors and zoonotic parasitic diseases and for predicting their spatial distribution. Including socioeconomic and host animal factors in epidemiological models to increase the accuracy of their predictions will help further develop and improve the field of environmental epidemiology.

## Supplementary Information

**Additional file 1:** **Table S1.** Attributes of the natural environmental variables. **Table S2.** Timing of the epidemiological survey and collection of natural data.

## Data Availability

All the data generated or analysed during this study are included in this published article.

## Abbreviations

AE: Alveolar echinococcosis
Aut: Autumn
CE: Cystic echinococcosis
CultivatedR: Proportion of cultivated land to total land use area
DEM: Digital elevation model
DIC: Deviance information criterion
ForestR: Proportion of forest to total land use area
GrassR: Proportion of grassland to total land use area
Max: Maximum
Min: Minimum
NDVI: Normalized difference vegetation index
Pre: Precipitation
Rh: Relative humidity
RMSE: Root-mean-square error
RPD: Residual predictive deviation
Spr: Spring
STAR: Structured additive regression
Sum: Summer
Sun: Sunshine duration
T: Temperature
Win: Winter
