# Supplementary material for: The impact of environmental factors on human echinococcosis epidemics: spatial modelling and risk prediction
Source: Parasit Vectors. 2022 Feb 8;15:47. doi: 10.1186/s13071-022-05169-y (PMC8822772; doi:10.1186/s13071-022-05169-y)
Supplement: Supplementary file 1 — Additional file 1: Table S1. Attributes of the natural environmental variables. Table S2. Timing of the epidemiological survey and collection of natural data. [file 13071_2022_5169_MOESM1_ESM.docx]

**Table S1.** Attributes of natural environmental factor variables.

| **Category** | **Factor** | **Description** | **Variable** |
| --- | --- | --- | --- |
| Climate | T | Temperature | T_Min, T_Mean, T_Max, T_Spr_Min, T_Spr_Mean, T_Spr_Max, T_Sum_Min, T_Sum_Mean, T_Sum_Max, T_Aut_Min, T_Aut_Mean, T_Aut_Max, T_Win_Min, T_Win_Mean, T_Win_Max |
|  | Pre | Precipitation | Pre_Min, Pre_Mean, Pre_Max, Pre_Spr_Min, Pre_Spr_Mean, Pre_Spr_Max, Pre_Sum_Min, Pre_Sum_Mean, Pre_Sum_Max, Pre_Aut_Min, Pre_Aut_Mean, Pre_Aut_Max, Pre_Win_Min, Pre_Win_Mean, Pre_Win_Max |
|  | Rh | Relative humidity | Rh_Min, Rh_Mean, Rh_Max, Rh_Spr_Min, Rh_Spr_Mean, Rh_Spr_Max, Rh_Sum_Min, Rh_Sum_Mean, Rh_Sum_Max, Rh_Aut_Min, Rh_Aut_Mean, Rh_Aut_Max, Rh_Win_Min, Rh_Win_Mean, Rh_Win_Max |
|  | Sun | Sunshine duration | Sun_Min, Sun_Mean, Sun_Max, Sun_Spr_Min, Sun_Spr_Mean, Sun_Spr_Max, Sun_Sum_Min, Sun_Sum_Mean, Sun_Sum_Max, Sun_Aut_Min, Sun_Aut_Mean, Sun_Aut_Max, Sun_Win_Min, Sun_Win_Mean, Sun_Win_Max |
| Geographical landscape | DEM | Digital elevation model | DEM_Min, DEM_Mean, DEM_Max |
|  | NDVI | Normalized difference vegetation index | NDVI_Min, NDVI_Mean, NDVI_Max, NDVI_Spr_Min, NDVI_Spr_Mean, NDVI_Spr_Max, NDVI_Sum_Min, NDVI_Sum_Mean, NDVI_Sum_Max, NDVI_Aut_Min, NDVI_Aut_Mean, NDVI_Aut_Max, NDVI_Win_Min, NDVI_Win_Mean, NDVI_Win_Max |
|  | GrassR | Area proportion of grassland in total land use | GrassR |
|  | ForestR | Area proportion of forest in total land use | ForestR |
|  | CultivatedR | Area proportion of cultivated land in total land use | CultivatedR |

**Table S2.** Time of epidemiological survey and natural data.

| **Province** | **Number of counties** | **Year** | | |
| --- | --- | --- | --- | --- |
|  |  | **Epidemiological survey** | **Natural data for analysis** | **Natural data for prediction** |
| Inner Mongolia | 16 | 2012 | 2012 | 2016 |
| Ningxia | 19 | 2012 | 2012 | 2016 |
| Gansu | 56 | 2012 | 2012 | 2016 |
| Qinghai | 39 | 2012 | 2012 | 2016 |
| Sichuan | 35 | 2012 | 2012 | 2016 |
| Yunnan | 5 | 2012 | 2012 | 2016 |
|  | 19 | 2016 | 2016 | 2016 |
| Xinjiang | 81 | 2012 | 2012 | 2016 |
| Tibet | 4 | 2012 | 2012 | 2016 |
|  | 70 | 2016 | 2016 | 2016 |
